# Financial Inclusion Paradigm Shift in the Postpandemic Period. Digital-Divide and Gender Gap

**DOI:** 10.3390/ijerph182010938

**Published:** 2021-10-18

**Authors:** Valentina Vasile, Mirela Panait, Simona-Andreea Apostu

**Affiliations:** 1 Institute of National Economy, 050771 Bucharest, Romania; valentinavasile2009@gmail.com (V.V.); simona.apostu@csie.ase.ro (S.-A.A.); 2 Department of Cybernetics, Economic Informatics, Finance and Accounting, Petroleum-Gas University of Ploiesti, 100680 Ploiesti, Romania; 3 Department of Statistics and Econometrics, Bucharest University of Economic Studies, 010552 Bucharest, Romania

**Keywords:** financial inclusion, digitalization, financial education, corporate social responsibility, principal component analysis, cluster analysis

## Abstract

Financial inclusion is strongly differentiated by age groups and countries and the pandemic has highlighted the increased gaps and inequalities but also the weaknesses of the system, in terms of flexibility, access and facilities of the customer-bank relationship and also from the perspective of the financial education of young generations and vulnerable people, active in the labor market. Based on the available data provided by the Global Findex database, and some findings after more than one year of COVID-19 crisis we outlined the main aspects of financial digitization, by categories of people and countries. At the same time, we identified the challenges and problems during the pandemic that significantly adjusted the consumption pattern of citizens and increased the need for on-line access for financial transactions. Starting from the analysis of the inequality of access to financial instruments in the last years, from the informational asymmetry in financial education and the challenges of the pandemic period, we underlined the main coordinates of changing the model of sustainable financial inclusion—based on five pillars—access, education, support tools, CSR and resilience. The research results highlight the need for convergence in providing opportunities to consider financial inclusion as a public good and an active tool to increase consumers’ satisfaction and the quality of life of individuals.

## 1. Introduction

Financial inclusion is currently a facilitator for growth and resilience. It is closely linked to seven of the all 17 UN SDGs, i.e., 1, 4, 5, 8 9, 10 and 12 ones. An inclusive financial system (accessible and operational) is an essential infrastructure in every country and an accelerator of quality of life, both through the contribution of education, facilitating financial digitization, streamlining leisure time and expanding access to funds for individual and household development. According to World Bank experts, financial inclusion “is a key enabler to reducing poverty and boosting prosperity” [1,2] building confidence and economic growth [3] and social resilience [4]. Financial inclusion access asymmetry is more evident in less developed countries that face a diversity of barriers—a significant informal sector, regional divides, gaps in access to education and employment (gender, age groups, poverty, etc.). That is why financial inclusion is perceived as an important factor in bridging these divides, and for supporting better economic and social outcomes. Financial inclusion is a major concern not only for credit institutions and public authorities from the perspective of attracting new consumers and launching affordable products but also for international organizations. The Financial Action Task Force (FATF) has an important interest in financial inclusion taking in account the “objective of protecting the integrity of the global financial system, which requires covering the largest range of transactions that pose money laundering and terrorist financing risks” [5]. Financial inclusion is multidimensional, given the diversity of challenges faced by different categories of stakeholders under the impact of phenomena such as terrorism. So, financial inclusion is defined by Financial Action Task Force as “ensuring access to appropriate financial products and services at an affordable cost in a fair and transparent manner” [5].

The COVID-19 crisis has generated not only challenges but also opportunities in the financial field, because lockdowns and social distancing measures have impact on the activity of credit institutions and their clients’ behavior. On the one hand, consumers encountered difficulties in repaying bank loans due to declining incomes and inability to travel, but on the other hand, electronic transactions were favored to the detriment of cash payments, with positive effects on financial inclusion [6]. In fact, digital financial services were during the lockdown period almost the only option for customers to do financial operations and shopping [7]. Therefore, the COVID-19 crisis (a black swan event) generated the intensification of the process of digitization of financial inclusion [8,9]. A new era of digital financial inclusion was opened by COVID-19 crisis, but the artificial intelligence and fintech are essential factors in this process [9,10].

The COVID-19 crisis has generated extensive social policy measures by public authorities targeting both the population and companies with an impact on financial inclusion. Thus, social assistance payments for populations have generated the opening of accounts in the conditions of minimal or zero physical contact but which takes into account the rules against money laundering and terrorism [11,12,13,14]. So, the pandemic period reiterated the need for a wide expansion and digitalization of financial inclusion, including for the traditionally conservative or with limited access population—from rural areas, day laborers, the elderly, people from poor households, etc.

The launch of the SDGs in 2015 generated intense and diversified implication from international institutions and researchers. Some experts concluded that the efficient management of financial resources at the micro- and macroeconomic level may become one of the keys to secure the funds needed to achieve UN-SDGs 2015 goals [15,16,17,18,19,20]. From this perspective, “provision of financial services at affordable costs to the disadvantaged and low—income segments of society” [21] ensures the inclusion in the official financial circuit of vulnerable categories, for which economic decisions have a dramatic impact on their existence and consumption profile. Contracting a real estate loan for a period of 30 years, for example, can have a major impact on the financial situation of a young person, especially in conditions where crises multiply and affect the income received by the employees.

Reducing the risk of poverty, intensifying the process of saving and investing at the level of individuals, the possibility of setting up and financing business, reducing the asymmetry of information on the financial market, establishing alternative job opportunities, feeding entrepreneurship, increasing the resilience of households to shocks are just some of the positive externalities of increasing financial inclusion by using a wider variety of specific products and services [22,23,24,25,26,27,28]. The higher participation of the population on the financial market is mainly due to the increase of the living standard but, recently, was pushed up by pandemic restrictions (lockdown, limited activities for consumers or on-line transactions, etc.). Higher incomes generate also an increased propensity for consumption which is fueled by the offers of traders and financial institutions [29,30,31]. On the other hand, “democratization of credit” or “easy credit culture” has led to a significant increase in the share of the population accessing credit through various channels, registering the excessive indebtedness of certain categories of people, generally those with a low degree of financial education and low income earners. The widespread use of mobile phones by the population has also led to the emergence of specific financial services such as mobile banking, mobile payments, money transfers, and mobile international remittance services [19,27,31,32,33]. So, financial inclusion is a complex process that is also fueled by the involvement of public authorities, financial institutions, IT companies that come up with solutions for the digitization and secure of financial services. In addition, the attitude of consumers towards transactions’ digitalization is important in the process of financial inclusion increase.

Financial inclusion has regional peculiarities, which is why the authors of this paper focused their analysis on European countries, given the existence of common or similar regulations in these states, a single market for goods and services, with transnational financial companies operating in most countries. The analysis conducted by the authors is distinguished by the methods used and the clustering of European countries, which reveals a direct link between the degree of financial inclusion and the level of development of a country. The phenomenon of financial inclusion is approached in the context of numerous influencing factors, related to the behavior of consumers who have lost confidence in the banking system, the COVID-19 crisis that generated the acceleration of digitalization of financial operations, increasing social responsibility of credit institutions and the need to promote business ethics.

The aim of the research is to highlight the dynamics of postpandemic financial inclusion and change the modality of using bank accounts by the population in the last decade and to estimate the effects of forced financial digitization following the COVID-19 crisis. For this, we have defined specific objectives:

1. Identifying the factors that influence the increase of access to use digitization facilities for payments and savings.
2. Analysis of the evolution of bank account holders, as the main form of facilitating the expansion of current online payments, respectively the degree of banking of the payment system, and presentation of differences depending on various attributes—level of economic development, population categories, including gender approach.
3. Establishing the impact of the pandemic crisis on the demand and supply of online payments, respectively of financial inclusion, after a year of pandemic, and some estimates of the increase of postpandemic bank account holders.
4. Identifying the potential for increasing financial inclusion through companies’ social responsibility (CSR) in small and medium private companies in emerging economies.
5. Establishing the minimum coordinates necessary for the wide promotion of financial inclusion, as a pillar of the quality of life after the pandemic. Starting from the analysis of inequality of access to financial instruments in recent years, of use of specific products/services, from the information asymmetry to support financial inclusion and from the challenges of the pandemic period, we identified the main coordinates of changing the model of financial inclusion—based on five pillars—access, education, support tools, CSR and resilience.

Therefore, the paper is structured as follows. The literature review presents an overview of the most important studies regarding financial inclusion, while Section 3 is dedicated exploring the implications of financial inclusion using a bibliometric analysis. Section 4 incorporates information related to data and methods used in the analysis. Section 5 presents the main empirical results, while the last parts highlighted the main discussions and conclusions of the research.

The findings highlighted the need for convergence in supporting financial inclusion as a public good, in providing opportunities to use financial inclusion as a tool to increase the quality of life of individuals and convergence between countries, areas of residence and groups of individuals.

## 2. Literature Review

The population’s attitude towards the financial sector is in a process of significant changes considering the effects of the two crises that marked the beginning of this century.

On the one hand, the international financial crisis generated a decrease in public confidence in the financial system given the failure of the banking market due to the exacerbation of the use of securitization operations and the launch of securitized financial products, very complex, difficult to evaluate even by rating agencies. The decline in confidence was also fueled by financial scandals involving large banks such as Societe Generale but also regional crises such as the Swiss franc crisis in Central and Eastern Europe [34]. Credit institutions have shown a corporate social irresponsibility (CSIR) that has primarily affected their own customers, but the devastating effects of the international financial crisis have been felt throughout the world economy. This concept of CSIR has gained consistency in the literature [35,36,37] given the numerous scandals fueled by the corruption and greed of managers of large transnational corporations. This concept has an extended applicability in the banking system considering the characteristics of the financial market (asymmetry of forces between the bearers of demand and supply) but also the traded of dematerialized assets. So, in the case of credit institutions, CSIR is fueled primarily by the information asymmetry that characterizes this market in which consumers have a low level of financial education and face inequities from banks such as abusive contractual clauses, misleading promotional techniques, bank frauds. Conversely, the companies take into account their social involvement and aspects related to the development of local communities, increasing the level of financial education of employees, supporting educational or health units [37,38,39]. In order to increase consumer confidence, more and more financial institutions have been involved in promoting the principles of sustainable development by launching CSR programs aimed primarily at local communities or improvement of financial education of their clients. Financial institutions are reshaping their business strategy given the importance of consumers’ force who have begun to know their rights and who are more informed and educated [38,39,40,41,42,43,44,45]. So, CSR in the financial sector involves shifting interest from maximizing profit for shareholders to increasing value for stakeholders, the most important of which are customers and shareholders. Given the complexity of financial products and services but also the multitude of financial decisions throughout lifetime of the individuals, some of its with long-term effects, better financial education programs are usually promoted in partnerships between financial institutions, market authorities, schools and universities, and even companies (the latter being aware of the importance of financial well-being on employee productivity at work but also the need to build investment saving plans for the retirement period). Financial education, usually, focuses on vulnerable or potentially long-term inactive categories such as women and adolescents and aims to generate a rational saving and investment behavior, given the process of financial innovation, population aging and intensified investment frauds [46]. However, the effectiveness of financial education programs is limited, with the age of targeted consumers being essential. Lusardi et al. [47] consider that “They are more effective when targeted at peak saving years (e.g., after age 40)”.

On the other hand, the current health crisis has determined the repositioning of consumers towards financial institutions, through the widespread use of internet or mobile banking services at a time when expressions such as physical distance and lockdown were the order of the day. The specialists noted that the digitalization phenomenon has a considerable impact on consumers, public institutions and companies, which is why it is considered to have a predominantly positive contribution on the socioeconomic development. Digitization generates challenges but also opportunities for various categories of stakeholders, both for the demand and supply bearers of financial products and services [32,48,49,50,51,52]. On the financial market, digitalization has had a rapid implementation considering the specifics of the traded services and products. Technical progress has generated a digital financial ecosystem that has definite advantages such as low cost of transactions, speed, flexibility that thus eliminates the constraints of the cash economy. In the banking sector, the very strong competition between local banks but also that generated by the branches of foreign banks have determined an increasingly intense race to attract new customers (red queen race), which is also manifested in terms of digitization [53].

The promotion of digitalization in the financial sector is also generated by the opportunities offered by technology in the fight against informal economy, money laundering and terrorist financing [54,55]. However, the expansion of digitization is hitting certain limits, especially in the banking sector because of concerns regarding clients’ privacy (banks have access to a lot of information that can be used, in certain circumstances, in a not very ethical way) and security of the transactions and data. In addition, the lack of skills among the stakeholders for using digital services is an impediment to the expansion of financial market digitalization for older people or those with a lower level of education having reduced digital skills. 

Concerns regarding cybersecurity in the financial sector have increased considerably in recent years given the extensive use of information systems in conducting day-to-day operations, but also the interdependencies specific to this field of activity that brings together not only financial institutions but IT firms, consulting companies etc. Moreover, the consequences of cyberattacks would be devastating and would “endanger the provision of liquidity by central banks and jeopardize the implementation of monetary policy”, loss of funds and intellectual property, and reputational risk for financial institutions [55,56]. During the pandemic, cyberattacks intensified in the financial sector, being the area most affected by this phenomenon. For this reason, the cyberattack is considered a component of the operational risk of financial institutions. Important investments have been put into the security of information systems and in human resources that know how to properly manage applications, devices and their information [57].

Another factor that limits access to digital financial services is the low level of consumer income. Some consumers cannot afford sophisticated devices to ensure their participation in the digitalized financial market. So, there is a gap between the offer of financial institutions and the technical possibilities of consumers, especially those in vulnerable categories. Internet access is another aspect that generates problems in ensuring a certain degree of financial inclusion given the reorganization of the financial system under the impact of digitalization (many branches or offices of banks have been closed, thus making the transfer from traditional services to those online). So, the digital divide is a reality of the current financial system, fueled by economic, social, technological and psychological factors [19,58,59].

Despite the expansion of online payments, there are specialists who demonstrate the importance of maintaining cash in the process of financial inclusion considering that “cash is still a fundamental aspect of the armory for financial inclusion” [51]. In the case of the poorest and most marginalized parts of society, cash is the only option for payments to meet the needs and fulfill certain financial obligations. From this reason, even during the pandemic, despite the recommendations for payment by bank card, the authorities did even issue concrete regulations by which credit institutions were obliged to provide cash services.

The current crisis has demonstrated the importance of digital financial services, but we cannot talk about the generalization, given certain limitations generated by technical infrastructure, consumers’ ability to use various applications and platforms to access the financial market, limited use of certain banking products or limited access generated by poor financial education. For these reasons, online banking penetration rates vary greatly, including between EU member states, with notable differences between Western countries and new members such as Bulgaria or Romania [60].

So, the process of financial inclusion involves concerted efforts both on the vulnerable beneficiaries and part of the stakeholders, i.e., with CSIR. In addition, the digitalization process generates many challenges for stakeholders, and the COVID-19 crisis has intensified both the concerns of financial institutions for providing safe and accessible services and consumers care for online products and services. Basically, we faced a paradigm shift towards accepting digital financial inclusion but also in the business model, towards affordable and diversified financial services and banking products. The development of financial infrastructure is not the only solution to increase the degree of financial inclusion. In addition to the efforts of the authorities with supervision, regulation and control of the financial market and financial institutions, financial inclusion should also be based on the knowledge of consumers who must be able to make good decisions, given the multitude of financial products and services that often are nonstandardized (which increases the difficulty of the choices made). For this reason, more and more scientific studies have shifted the interest from the analysis of the supply side of financial markets to the role played by the demand that comes from financial consumers. The existence of well-educated clients from a financial point of view reduces the information asymmetry specific to this market [61,62,63,64,65].

Through the bibliographic selection and the associated comments, we have delimited the main factors that influence the financial inclusion, and their diversity and complexity are ascertained. The dynamics of postpandemic financial inclusion and the capitalization of some of the adaptive behaviors depend, on the one hand, on the degree of readiness and stage of prepandemic financial inclusion and, on the other hand, on the consumer response to the restrictions imposed during the crisis. The pandemic is in its fourth wave, and individuals and the business environment are redefining the coordinates of their postpandemic life, among which, financial inclusion is configured as a pillar of rebalancing economic, social and societal robust recovery and future sustainable development. The empirical analysis shows where we started, some evolutions in the first year of the pandemic, and in the comments we will outline the proposed model of sustainable financial inclusion, postpandemic, from the paradigm shift generated by digital divide and increasing gender gaps.

## 3. A Bibliometric Analysis on Financial Inclusion

In order to consider in our state of the art the most relevant studies in the field, we used the bibliometric analysis, the principal source of scientific articles being the academic platform Web of Science.

First, we explored the content of the first 250 research articles related to financial inclusion in terms of the number of citations. In order to highlight the structure of the scientific field, we used content analysis, inspecting the most common words and the relationship between words. Co-occurrences with a frequency of at least 10 times were taken into account, with a correlation degree greater than 0.5. The analysis was done using the Vos program.

**Figure 1:**
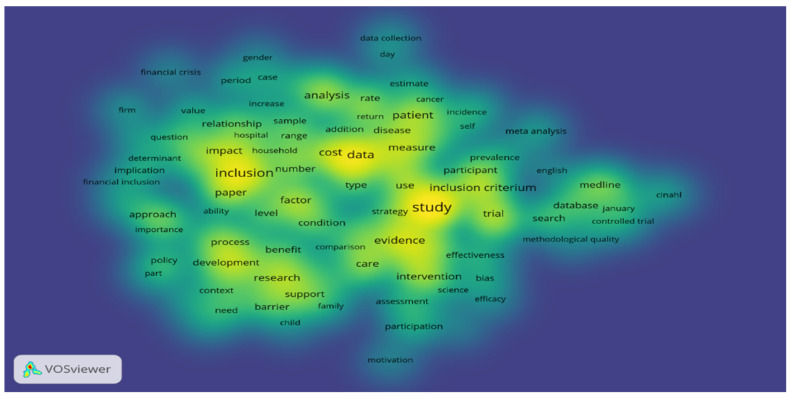
Most common words in scientific publications regarding financial inclusion.

The empirical analysis proved that the most common words in the full content of selected articles apart of the keywords used are: “inclusion”, “study”, “cost”, “data”, “analysis”, “patient”, “evidence”, “intervention”, “inclusion criterium”, “measure”, “factor”, “research”, “impact”, “development”, “care”, “support” (Figure 1).

**Figure 2:**
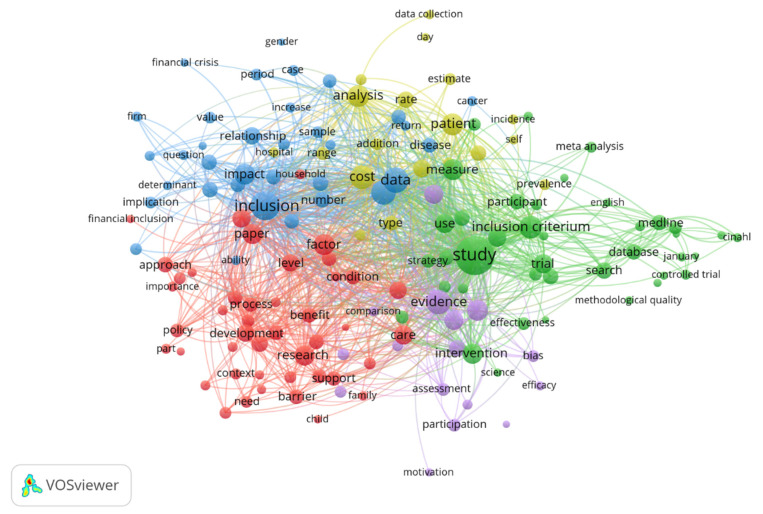
Word network in economic scientific publications’ content.

The most common word combinations identified in the most relevant studies related to financial inclusion are process-development-approach-importance-policy-condition, inclusion-impact-determinant-implication, analysis-patient-sample-cost-type, study-inclusion-criterium-trial-database-trial, participation-efficacy-assessment-bias (Figure 2). In order to identify the combination of words the most often encountered, we explored the most correlated words within the selection of articles, using as threshold the value of 0.5.

## 4. Data and Methodology

In the financial domain, clients are heterogeneous, with different expectations and behavior [66]. Thus, the most useful technique for analyzing consumer behavior in the financial field is customer grouping. In this way, customers are divided into homogeneous clusters in terms of needs and characteristics [67].

Customer grouping can be achieved depending on the characteristics: profitability [68], customer behavior [67,69], the degree of customer loyalty [70,71,72,73,74,75]. Wang et al. [76] clustered clients using language variables. Newstead and D’Elia [77] showed that grouping customers based on the color of their vehicles is very important in the field of insurance. Using the clustering method, Zeithaml et al. [68] introduced the concept of customer pyramid through which they are divided according to customer profitability. Neal [70] segmented customers according to their characteristics and consumption. Ansari and Riasi [66] clustered the banks’ clients according to demographic and financial characteristics.

The large number of credit link variables leads to a more difficult and complicated data analysis. Therefore, highly correlated variables are grouped using mainly component analysis [78]. With the help of the analysis of the main components a large number of variables is reduced to a smaller set of main factors which summarize the essential information contained in the variable [79].

To understand the behavior of account holders, Ringim and Yussof [80] conducted research using several variables on perception and awareness, of which they are the most important using the analysis of the main components. The analysis of key components was used by Bruce et al. [81] to assess performance in the internet banking industry, by Adler et al. [82] for replacing input/output parameters, and by Shanmugam et al. [83] for classifying units. Yaghoubi and Bashiri [84] used the analysis of key components to determine the effectiveness of high-risk units within Iranian bank branches.

Based on the previous theoretical considerations, in order to determine the factors influencing holding a bank account of the European citizens the following three hypotheses have been formulated:

**Hypothesis** **1** **(H1).** *There are certain factors (gender, age, education, incomes, area, and employment) that influence holding a bank account.*

**Hypothesis** **2** **(H2).** *There are differences between groups/countries in terms of holding a bank account.*

**Hypothesis** **3** **(H3).** *Economic well-being and level of education explain the differences between countries related to holding a bank account.*

In order to answer the hypotheses, we used the data provided by Global Findex [85], the world’s most comprehensive data set on how adults save, borrow, make payments and manage risk. The data provided by Global Findex are collected through nationally representative surveys, including over 150,000 adults and over 140 countries.

The variable account represents the percentage of respondents who report having an account (by themselves or together with someone else) at a bank or another type of financial institution. This variable is split according to gender, level of education, age, origin and standard of living.

The socioeconomic variables are provided by Eurostat [86]. The period analyzed is 2010–2020 and the statistical software tools used for analysis were SPSS and Tableau.

Gross Domestic Product is calculated at market prices, chain linked volumes, index 2010 = 100.

The variable describing the level of education reflects five variants: primary education, secondary education, bachelor’s degree or equivalent level, master’s and doctoral studies.

The methods used were average comparisons, principal component analysis, and cluster analysis.

In order to select the variables that most influence holding a bank account we used the principal component analysis.

Principal component analysis (PCA) is a multivariate analysis technique for a data table in which observations are described by quantitative dependent variables. Its purpose is to extract important information from the table so that it can be represented as a set of new orthogonal variables called principal components. Thus, the similarity pattern of observations and variables is displayed as points in maps [87].

The visualization and statistical analysis of the principal components contribute highlighting the similarities and differences between the samples and the importance of the original variables on the first components [88]. Principal component analysis of a data matrix extracts the dominant patterns in the matrix in terms of a complementary set of score and loading plots [89].

The purpose of PCA is to condense the information of a large set of correlated variables into a few variables, while not throwing overboard the variability present in the data set [90].

Several procedures have been proposed for determining how many principal components to retain: the Kaiser rule and the scree plot (Cattell), resampling methods, cross-validation methods, Bayesian procedures and statistical tests. These statistical tests assume that variables are not standardized before carrying out the PCA [91].

Forkman and Piepho [92] proposed testing these null hypotheses sequentially, starting with ***K*** = 0 and continuing with ***K*** = 1, 2,…, until a nonsignificant result is obtained or ***K*** = ***M*** − 2.

For testing the null hypothesis, Yochmowitz and Cornell [93] proposed to use the statistic:

(1) $$T=\frac{{\hat{\tau }}^{2}*K+1}{\sum_{k=K+1}^{M}{\hat{\tau }}^{2}}$$

as long as ***K*** < ***M*** − 2. The sequential testing procedure ensures the level of significance conditionally on the null model and protects against overfitting [92].

Several procedures have been proposed for determining how many principal components to retain: the Kaiser rule and the scree plot (Cattell), resampling methods, cross-validation methods, Bayesian procedures and statistical tests. These statistical tests assume that variables are not standardized before carrying out the PCA [93].

The weakness of PCA is it constructs principal components without considering the reference information [94].

The advantages are:

- The way PCA separates information and reduces the size of the data;
- That PCA is a versatile and handy method [95]:
- That PCA is a powerful technique for data transformation;
- That this method is useful wherever high-dimensional data sets are encountered it before further analytical work.

To classify the countries according to holding a bank account we used cluster analysis.

*Cluster analysis* sorts data vectors into homogeneous groups when adhesions to true clusters are unknown. For grouping are using similarity matrices or the distance between individual vectors and vector groups [95].

Cluster analysis assumes grouping similar observations in homogeneous subsets, revealing patterns related to the studied phenomenon. To evaluate if exists a similarity between objects, we used a remote function and a wide variety of grouping algorithms. First we calculated similarity measures between observations and once the observations are grouped into clusters we calculated similarity between clusters [96].

Grouping methods come in four types: hierarchical, partitioning, overlaying, and ordering algorithms. The method determines the capacity of the cluster configuration recovery methods existing in the data, thus validating the algorithms [97].

Sokal and Michener [98] developed group correlation matrices, the correlation method representing the foundation of the hierarchical grouping algorithm. The objective of the hierarchical grouping algorithm is represented by a dendrogram, grouping all the elements into a single tree. A node is created to join two or more elements, calculating a node expression profile by averaging the integrated elements [99].

Hierarchical cluster analysis is a method used to identify the underlying structure of objects through an iterative process that associates (agglomerative methods) or dissociates (divisive methods) object by object, being halted when all objects have been processed [100].

The agglomeration procedure begins with each object in a separate cluster and then combines the sequences in sequence, reducing the number of clusters at each step, until all objects belong to a single cluster [101].

Cluster analysis identifies and groups all variables into a small number of homogeneous groups. The beginning of the analysis consists in separating the variables into a clear vision. In each step the classes that are homogeneous are grouped, the final result being a cluster that will contain all the analyzed variables. Distance analysis is the basic criterion by which the selection is made. The side objects will belong to the same group, while the variables that have large distances will be divided into different clusters.

To identify intergroup similarity we used first single-linkage or the similarity of the closest pair:

dSL(A, B) = mini ∈ A, j ∈ Bdi,j(2)

Then we used complete-linkage or the similarity of the furthest pair:

dCL(A, B) = maxi ∈ A, j ∈ Bdi,j(3)

Third we used group-average or the average similarity between groups:

dGA = 1NANB∑I ∈ A∑j ∈ Bdi,j(4)

In order to explain the differences between countries related to holding a bank account we used ANOVA. The analysis of variance test (ANOVA) has long been a very useful tool in studies on several experimental groups and one or more control groups, but cannot provide detailed information on the differences between different study groups or on complex combinations of studies [102].

ANOVA examines the differences between the means of two or more independent groups [103]. Similar to Welch’s *t* test, Brown-Forsythe’s F statistic [104] can be calculated using individual group variances as follows:

(5) $$F=\frac{\sum_{j=1}^{k}n_{j}*{\left(M_{j}-M\right)}^{2}}{\sum_{j=1}^{k}\left(1-\frac{n_{j}}{N}\right)s_{j}^{2}}$$

where ***N*** is the size of the total sample, ***k*** refers to the number of groups compared, $M_{j}$ and $s_{j}^{2}$ are the mean of the sample and the variance of group ***j***, respectively, and *M* is the mean of the population.

## 5. Results

### 5.1. Dynamics of Financial Inclusion in the Last Decade, Based on Transaction Banking and Digitization, Using the Findex Database

**Figure 3:**
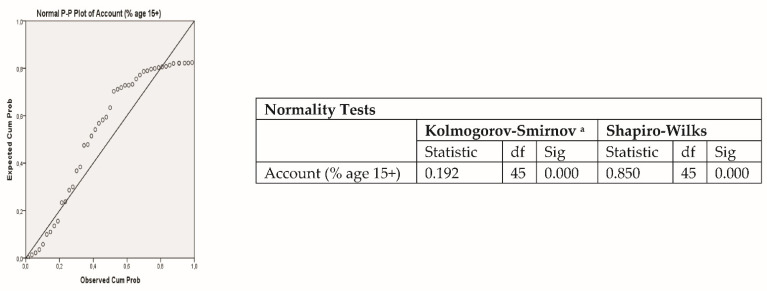
Normality of owning an account variable. ^a^ Lilliefors Significance Correction.

The variable owning an account follows an approximately normal distribution at the European level, as can be seen from the normality plot, confirmed by the Kolmogorov–Smirnov test (Figure 3).

**Figure 4:**
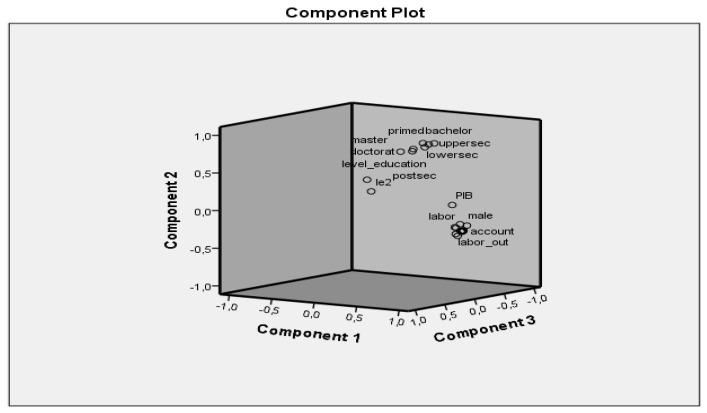
Component plot.

As can be seen from Figure 4, the variables on education are on a different component, and if we eliminate these variables there remains only one main component that explains the holding of a bank account (Table 1).

According to Table 2, the share of men with a bank account is 83.58%, and the share of women with a bank account is 80.89%. The share of men is approximately 3 p.p higher than the share of women, and this is also explained by the fact that the share of men who work is higher than that of women with job. Regarding their employment, 73.18% of those who do not work have an account, and of those who work, the share of bank account holders is 87.28%.

In terms of age, 67.1% of young people (25–24 years) own a bank account, and among adults (over 25 years) 84.94% own a bank account. This difference of over 17p.p. is explained by the fact that the youngest do not have a job, leading to the conclusion that having a bank account is the consequence of a formal employment relationship, most employees receiving money in a bank account, not cash. Moreover, having a bank account can lead also to a decrease in informal, undeclared work.

Another major difference is the level of education, among those with primary education 69.81% are holders of bank accounts, and among those with secondary education the share is 86.30%, as a person has a higher level of education. However, that person will have a better paid and declared job, so will have a bank account.

Regarding the standard of living, the share of the people registering higher incomes is wider than those with lower incomes (85.99% compared to 76.47%). The fact that those with lower incomes do not have a bank account can be explained by the fact that they can work unofficially, not receiving the salary in an account, so they do not need an account.

Eighty point sixty-seven percent of those living in rural areas, and 83.71% of those living in urban areas have a bank account, but the difference is not big, due to probably the fact that those who do not have a job in rural areas are searching for a job and working in its proximity, in urban areas.

Of all the variables analyzed, the biggest differences regarding the account holders are: age, education, standard of living and employment form. These differences are also highlighted by the tests between the groups, for all groups being registered a sig smaller than 5% (Table 3).

Therefore, gender, age, education, incomes, area, and employment form influence holding a bank account, confirming hypothesis H1.

**Figure 5:**
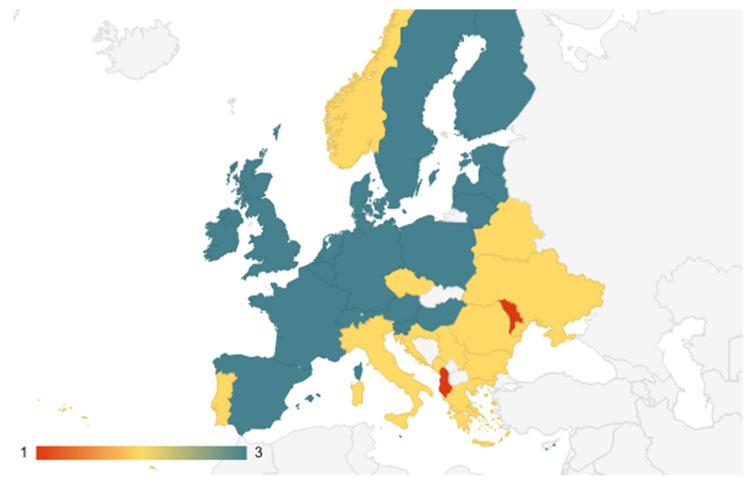
The clusters map.

Dividing the countries according to the analyzed variables resulted in three clusters:

- Cluster 1: Albania, Azerbaijan, Moldova;
- Cluster 2: Armenia, Bulgaria, Bosnia, Belarus, Georgia, Croatia, Macedonia, Montenegro, Romania, Russia, Turkey, Ukraine, Kosovo, Czech Republic, Italy, Norway, Portugal;
- Cluster 3: Austria, Belgium, Switzerland, Cyprus, Germany, Denmark, Spain, Estonia, Finland, France, UK, Hungary, Ireland, Israel, Lithuania, Luxembourg, Latvia, Malta, the Netherlands, Poland, Slovak Republic, Slovenia, Sweden (Figure 5).

To highlight the differences between the three clusters regarding owning a bank account, we performed ANOVA. According to the table, sig. < 0.05. Therefore, the variable owning a bank account explains the differences between the three clusters (Table 4), confirming the hypothesis H2.

In order to explain the differences between countries related to economic well-being and level of education, the ANOVA analysis was performed. The variable used to describe economic well-being is GDP/capita, and, for the level of education, the following variables were used to reflect all stages of education: primary, secondary, bachelor’s degree, master’s degree and doctorate. According to Table 5, the three clusters indicate significant differences according to GDP, instead the level of education does not lead to significant differences (Table 5), confirming also the hypothesis H3.

### 5.2. Aspects of Forced Digital Financial Inclusion in the First Year of the Pandemic. The Gaps Persist. An Analysis Using Eurostat Data

**Figure 6:**
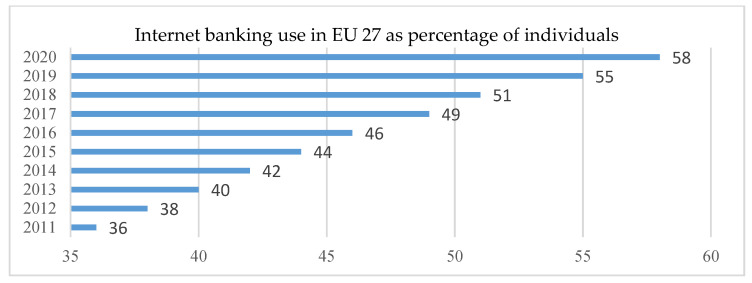
Internet banking—EU 27. Source: Authors projection using Eurostat database. Data extracted on 12 September 2021 online data code: TIN00099.

Over time, customers have changed banks only in extreme circumstances. Digitization has facilitated comparisons of banking services, cost and efficiency, and the restrictions imposed by the pandemic have reconfigured the relationship between the bank—small beneficiaries—individual customers or SMEs. Technological developments of digital tools supported new banking products and services such as a wide range of electronic payments, various payment portals and increased e-commerce. However, the percentage of banks customers that use online banking services is relatively low at European level compared to other markets. There are also significant differences in using banking services between EU member states and even between some regions (NUT3 level). While people in the Nordic countries use online services in overwhelming proportions, those in southern European countries are extremely skeptical about using such services. Financial inclusion is strongly correlated with the use of online banking systems. Thus, at EU level, in the period 2011–2020 internet banking, the main facilitator of digital financial inclusion registered an ascending trend, reaching in 2020 the highest value in this period (58%) (Figure 6). In 2020, the coronavirus crisis led to multiple changes. The first year of COVID-19 changed the structure of activities facilitated by the Internet to current utility (e-commerce, tax payments, etc., telework), imposing a “forced” financial education.

**Figure 7:**
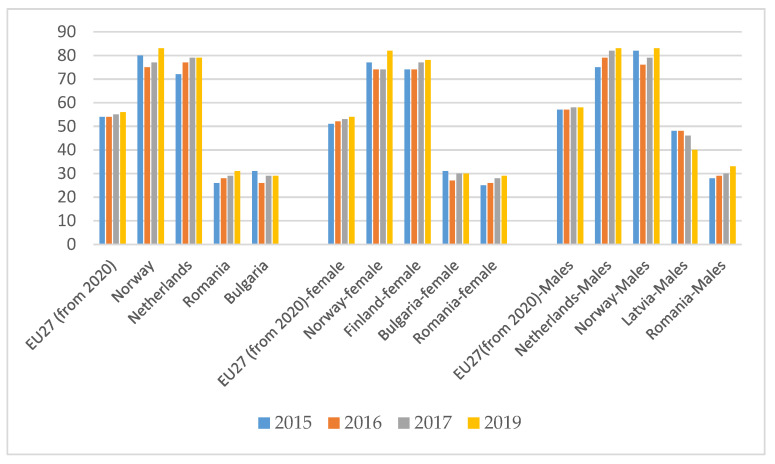
Individuals who have basic or above basic overall digital skills by gender as a percentage. Source: Authors projection using Eurostat database. Data extracted on 12 September 2021 online data code: TEPSR_SP410.

Until the time coronavirus crisis, about 50% of the population was not ready for online transitions, with men registering a higher level than women of basic or above basic digital skills. In Europe, for example, percentage of individuals (16–74 years old) who have basic or above basic overall digital skills (in the four specific areas—information, communication, problem solving, content creation) differs significantly by countries and gender, respectively from a minimum of 29% for women and 33% for men (Romania) to a maximum of over 80% for both genders in the Nordic countries. (Figure 7).

**Figure 8:**
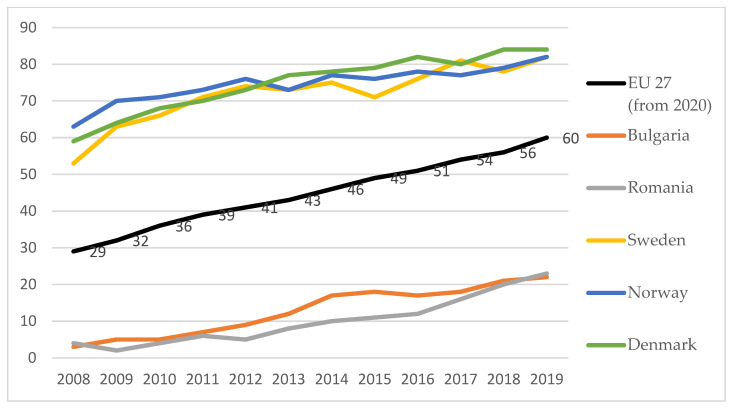
Individuals having ordered/bought goods or services for private use over the internet in the last 12 months, average EU 27 and extreme performance by countries as a percentage of individuals. Source: Authors projection using Eurostat database. Data extracted on 12 September 2021 online data code: TIN00096—Buy or order for private use. Within the last 12 months prior to the survey. Manually typed e-mails are excluded.

In terms of using the Internet in the last 12 months for ordering goods or services, the EU average level was lower than 30%, but over time it increased. In 2019 it reached almost 50% (Figure 8). Therefore, the year 2020 was even more a challenge at European level, the population not being accustomed and prepared for the online system, respectively financial.

**Figure 9:**
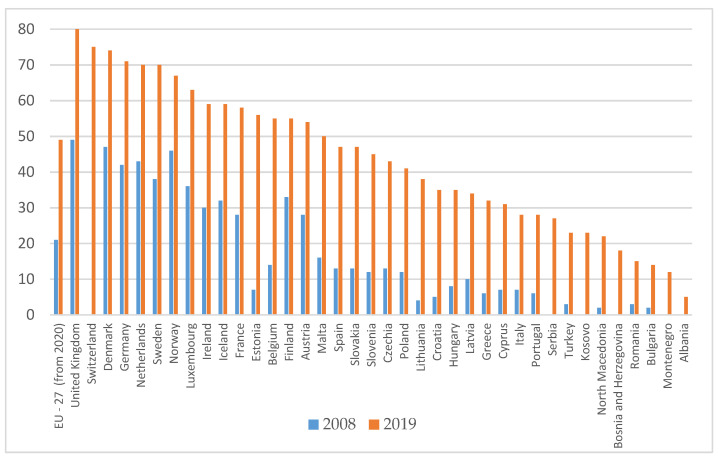
Individuals having ordered/bought goods or services for private use over the internet in the last three months, in 2008 and 2019—percentage of individuals. Source: Authors projection using EUROSTAT database, Digital economy and society (t_isoc_i). Data extracted on 12 September 2021, online data code: TIN00067.

In 2019, the best positioned countries in terms of internet use to order or buy goods or services for private use are: UK (80%), Switzerland (75%), Denmark (74%), Germany (71%), Sweden (70%), the Netherlands (70%). The lowest levels of use of internet ordering are in Romania (15%), Bulgaria (14%), Montenegro (12%) and Albania (5%) (Figure 9).

In addition to the population, the business environment was not well prepared for the transition to online or e-commerce, respectively. At the EU level, the pandemic affected traders of nonessential goods or services. The lockdowns severely restricted specialized shops’ activities. Moreover, the goods/services considered essential differ from country to country. Some countries considered only food and health essentials, while in other countries, the list of essential products included gardening, furniture and interior decoration or IT&C. E-commerce has thus become the lifeline for many traditional stores, which have had to quickly implement or expand online sales, click and collect shopping services or home delivery.

**Figure 10:**
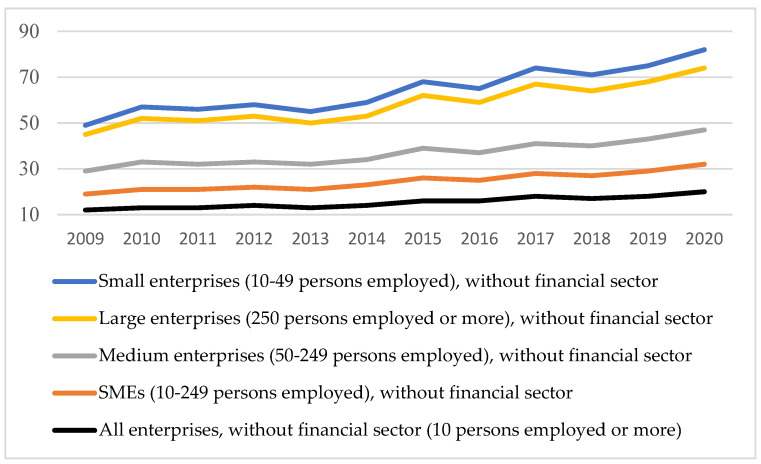
Share of enterprises’ turnover on e-commerce. Source: Authors projection using EUROSTAT database, Digital economy and society (t_isoc_i) Data extracted on 12 September 2021, online data code: TIN00110.

**Figure 11:**
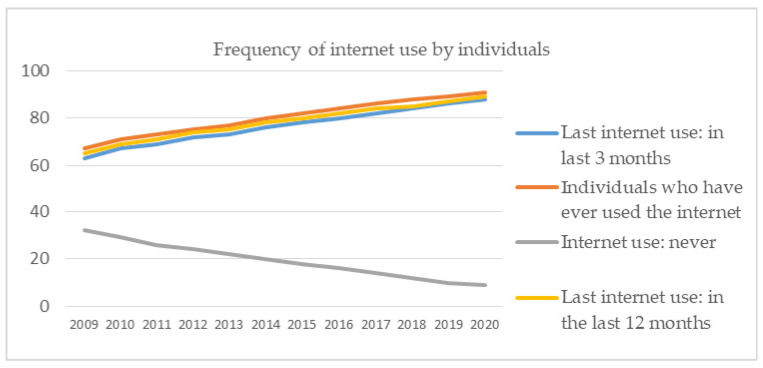
Frequency of internet use by individuals. Source: Authors projection using EUROSTAT database, Digital economy and society (t_isoc_i) Data extracted on 12 September 2021, online data code: TIN00028.

As can be seen from Figure 10, the share of enterprises’ turnover on e-commerce increased during the pandemic, regardless of the size of the company, because everyone had to switch to the online environment. The forecast on retail e-commerce sales as a share of retail trade in selected countries for 2021 indicated that the European average will decrease to 15.3% compared to 2020 (16.2%), probably starting from the premise that the crisis has already reached its peak. However, the value in 2020 is much higher than the values up to the time of the crisis. In 2019 it stood at 12%. Therefore, everyone has tried to adapt to the online system, with the share of individuals in the first year of the pandemic using the Internet increasing (Figure 11).

Specialized studies have revealed the importance of communication between financial institutions and consumers, the sites playing a decisive role in creating the image of banks among customers [43,105,106] Because of that “corporate website is shown to be a favorable tool for companies marketing specialists that can result in forming consumers’ positive perception-based bonds with the company” [107]. During the pandemic, the digitalization of communication between financial institutions and consumers has acquired new values [108,109,110], which is why we believe that in the future we can even talk about the digitalization of the financial education process.

Thus, the pandemic accelerated the digitalization of the whole society. The pandemic has had a positive impact also on the telecommunications market in terms of digitalization facilitating payments for a wider variety of goods or services. It has accelerated processes, but at the same time highlighted shortcomings. The result after a year of the pandemic is the digitalization of both the population and the companies, leading to financial inclusion and increased associated knowledge and skills, often gained through learning by doing. Another key factor for digitization was the development of the Internet: more broadband and a larger coverage area, especially in rural localities. 

## 6. Discussion

### 6.1. Some General Outcomes

Financial inclusion is one of the main challenges of the last decade, especially for less developed countries. The available amounts of money and the predominant model of money circulation (informal and cash-dominated economy versus cashless economy) on the one hand, and the confidence in the financial system, the development of the lending system for population and the digitalization of the client-bank relationship, on the other hand, adjusted the behavior of financial consumers and credit institutions [60].

The analysis of the phenomenon of financial inclusion reveals a very complex landscape at international and European level [19,111,112]. Even among EU countries, there are notable differences between member states, and a major cleavage can be detected between Western European countries and new member states. In the case of new EU member states, the reduced financial inclusion can be explained by the lower level of development of the financial system, poor financial education of population and lower incomes, with a higher share of workers paid in cash and not using a debit card for wages and salaries. Poor financial supervision in these countries has generated numerous frauds or regional crises that have further eroded consumer confidence in credit institutions or payments online. In addition, the international financial crisis in 2008 eroded consumer confidence in the banking system. The general perception is that credit institutions show a corporate social irresponsibility [29,30,37].

Regardless of the general level of financial inclusion in 2019, with major differences observed between different categories of consumers such as urban/rural, young/old, women/men or depending on the level of education, the pandemic restrictions reconfigured the importance of digital financial inclusion, in terms of both access to and use of the products. Therefore, differentiated measures are required to be implemented by credit institutions, public authorities with supervisory, regulatory and control responsibilities on the financial market, consumer protection authorities.

Financial inclusion is important from the perspective of the impact on economic growth and the mobilization of funds to support the achievement of the objects of sustainable development and, lately, human-centered recovery after pandemic.

Financial inclusion requires concerted efforts on the part of different categories of stakeholders. The presence of social responsibility on different levels is necessary. So, we have to consider:

- The social responsibility of corporations (banks) that carry out financial education programs and have an ethical behavior towards consumers. We have in mind the social ethics that aims to increase citizens’ access to digital financial services by attracting as many citizens as possible to the formal economy and the official banking system;
- The institutional social responsibility of central banks and consumer protection authorities, which creates the necessary legal framework for increasing citizens’ access to banking services and products and sanctions the unethical behavior of banks towards customers;
- The social responsibility of consumers who show openness to the accumulation of new knowledge and skills given the major changes in the financial market under the sign of financial innovation and digitalization. The need to increase the degree of financial inclusion should not turn into a favored factor for money laundering and terrorist financing. For this reason, the different categories of stakeholders involved (consumers, financial institutions, public authorities) have a common responsibility to create a sound framework, promote secure financial products and use banking products and services in good faith [15,17,21]. Promoting social responsibility can also take the form of partnerships (1) between credit institutions and companies to provide financial education programs for employees of large companies (these programs may target savings and investment programs to ensure financial comfort in old age, with retirement from professional life), and (2) between banks and educational institutions to lay the foundations of responsible financial behavior from childhood.

**Figure 12:**
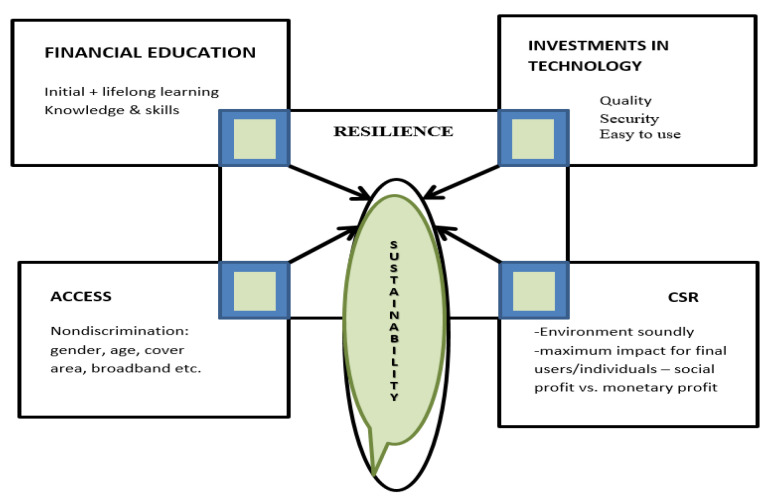
Sustainable digital financial inclusion proposed model for post-pandemic period. Source: Authors’ contribution based on research results.

So, the sustainable digital financial inclusion model in the postpandemic period is re-shaping as a result of the paradigm shift [113,114,115] of financial inclusion becoming a public good. Access, education, support tools/infrastructure, CSR and resilience are the main interconnected pillars of the new proposed model resulted from our research, considering the human-centered development after pandemic crisis [115] (Figure 12). The confidence of individuals in the use of banking system facilities (loans, savings products, etc.) and in the security of online transactions (e-commerce, payment of taxes, etc.) and low costs for such services are the foundations of robust recovery after the crisis and the starting point in creating tools to ensure the resilience of each pillar of financial inclusion and the sustainability of all together. Therefore, the sustainability of financial inclusion means not only growth and development of subsystems ensuring environmental protection components (ecoequipment, low risk of pollution, protection of bird habitats etc.)—i.e., digital devices or other specific equipment such as antennas, equipment factories, etc., but also the social responsibility of stakeholders to reduce risks that cause adverse effects—electronic fraud, inequity to customers, quality of services or discrimination in access, exacerbation of the role of monetary profit to the detriment social profit and customer loyalty to suppliers.

Therefore, the proposed model takes into account the following components of post-pandemic life:

- Digitalization will be accentuated insofar as the security of transactions increases and in the trust towards the transaction partners;
- The financial education of the individuals will change the business model of the banks and, in general, of the financiers or of the suppliers of goods and services, obliging towards social responsibility, in its various forms;
- Resilience will be the basic component in building the development strategies of companies and households;
- Discrimination in access will be reduced—whether it is discrimination of territorial area, costs, vulnerability (involvement of young people, women, low-income people, etc.).

Financial inclusion acquires new values in the context of various crises that have a major impact on the existence of individuals but also on the activity of companies and public authorities. The COVID-19 crisis has generated a rapid implementation of financial products and services used as social protection measures for the population. However, the rapid expansion of online payments and shopping is marked by fears about cyberattacks and the use of financial products to launder money and finance terrorism [6,8,9,12,13,14].

The cyber approach of the proposed model will ensure sustainability, and the development/promotion of components/mechanisms and tools for enforcing resilience will reduce the risks and impact of conjectural, extreme events such as the current pandemic crisis, prolonged by successive waves that obstruct the business environment and profoundly eroded/redesign the prepandemic life model. A “new normal” includes without question a model of financial inclusion with a growing digital component, and with protection/adaptation mechanisms specific to the concept of economic resilience (the return to performance indicators will be made in a way adapted/adjusted to needs and limitations of future development).

### 6.2. Theoretical and Practical Implications

The health crisis generated by COVID-19 requires a reconsideration of the phenomenon of financial inclusion, a series of theoretical and practical implications being identified. Starting from the analysis of the inequality of access to financial instruments in the past years, from the informational asymmetry in financial education and the challenges of the pandemic period, we underlined the main coordinates of changing the model of sustainable financial inclusion—based on five pillars—access, education, support tools, CSR and resilience. This model can be the starting point for the development of an index of sustainable resilient social inclusion in the postpandemic era that better reflects the dynamics of the phenomenon. Therefore, the proposed new model of financial inclusion can generate positive externalities not only on the banking market but can be the starting point for reconfiguring the instruments of social inclusion that must be under the sign of resilience and sustainability.

The stakeholders, like public authorities and credit institutions could use these ideas to reconstruct public policies and/or support business strategies. The reconfiguration of national financial inclusion policies is necessary in the context of the action of some determining factors with sometimes divergent impact, the main objectives being, at least, the following:

− Improving the digital competencies of consumers considering the separation of black swan type events (such as the COVID-19 crisis) which requires the use of home banking services;
− Modeling the behavior of consumers who face, on the one hand, the increase in the complexity of specific products as a result of the intensification of financial innovation and, on the other hand, with technical challenges generated by digitalization;
− Increasing the responsibility of credit institutions towards consumers who should be treated as equal partners, despite the asymmetry of information and financial power specific to the banking market;
− Limiting the threats posed by cyberattacks, money laundering and terrorist financing.

## 7. Conclusions

In this paper, we have presented some aspects of the financial inclusion highlighted by the pandemic as critical points in building the financial resilience of postpandemic individuals. Like any crisis, the pandemic generated both positive externalities (forced digitization including for individual and household financial transactions) but also negative ones (asymmetry of access to digital infrastructure necessary to switch from cash operations to online payments, increasing household income inequality due to the reduction of some economic activities). The analyses performed highlighted:

- Significant differences between countries from the perspective of the degree of banking transactions at the level of individuals, caused by both financial education and support infrastructure (access to digitization facilities, offer of financial services, cost of banked transactions, etc.).
- The preponderance of SMEs in the business environment and the CSR weaknesses at their level or the lack of concerns in this respect, with direct effects on the financial inclusion of employees.
- The main factors influencing the increase of access to the use of digitization facilities for payments and savings identified by the analysis were access to internet, level of studies, gender, employment characteristics and level of development.
- The limitation of some activities during the pandemic period reorganized the model of consumer transactions, both for individuals and for a large part of companies. After a year of pandemic, the bank account holders rescued, but limited, depending on the accessibility to the online banking system. Likewise, many small and medium-sized companies have digitized their collection and payment operations with the development of online commerce.
- The financial education in the pandemic represented especially a fortuitous information and options for adapting to online payments, according to the current offer, without the possibility of options based on rational choices of financial efficiency. Banks and companies have promoted their “adaptation” offers and the population has had few alternatives to choose from. Likewise, the employees of the small private firms, accustomed to cash payments in the relationship with the employer, were forced to switch to the use of debit cards, and for banking operations to access the online services of saving, payments, credit.
- From the perspective of promoting financial inclusion through CSR in SMEs we can identify some major changes at the company level, namely (a) greater use of social support tools for employees, e.g., accessing the financing of pandemic employment from special funds, developed at national level, flexibility of working time, use of banking instruments for payment of salaries, etc., and (b) computerization of production and distribution processes and hence change of job requirements including digital skills, use online communication devices, teleworking, etc.

Conducting financial education programs is a solution to reduce financial exclusion, thus it is necessary for specific programs to become increasingly complex and not consist only in transmitting basic knowledge of budget and investment. Consumers need skills in using devices taking in account digitalization of financial transactions. Therefore, financial education programs will be carried out by age groups, level of education, gender, to best meet the needs of financial information and learning digital skills for each group.

Consumer behavior is also key to increasing financial inclusion, including greater accountability in managing one’s own income, being able to overcome pressures and risks from traders and financial institutions that use misleading promotion techniques to sell products and services, including financial ones, and which even practice abusive contractual clauses (in the case of loans to individuals). Consumer behavior needs to be improved not only through financial education programs, but also by promoting consumer protection campaigns to raise awareness of their rights in the financial market. The social responsibility of credit institutions can acquire new “business values” by concluding partnerships with educational institutions so that banks have regular meetings with pupils and students and offer them financial education programs adapted to their level of understanding and financial need, because major differences exist for example between rural and urban areas. Even if these meetings involve certain expenses, in the long run, the results will be positive for banks through better knowledge of the demand and by gaining new clients. Therefore, accountability needs to become a priority for both financial institutions and consumers, given that poor financial decisions affect both the well-being and quality of life of customers and employees of financial institutions.

The health crisis has demonstrated the vulnerability of certain groups of people and raised an important issue, namely financial resilience. It is necessary for financial consumers to have a preventive behavior, in the sense of saving money every month, given that events such as the “black swan” can affect them at any time. The coronavirus crisis has accentuated the usefulness of financial digitization and raised new issues related to access to digitized financial instruments for all categories of citizens, again reiterating the need for transaction security and financial education of the population, as factors to facilitate digitization financial in the case of persons, for the financial flows generated by economic activities—banking the payment of salaries, incomes of entrepreneurs and those who carry out liberal professions, etc.—but also the development of the system of crediting persons for different activities—consumer loans, small investments etc.

The specialized literature has highlighted the barriers and opportunities of digitalization for economic and social development and for the reorganization of the consumption model of individuals. Moreover, the pandemic forced digitalization and, during this period, already about two years, it has reshaped the client–bank relationship through informal financial education and the changes will continue.

Unfortunately, the available databases—Global Findex database reflects the comparative situation between countries and categories of people only until 2017, but by processing the data, the existing gaps were clearly highlighted. The partial data available for 2019 and 2020 clearly demonstrate a paradigm shift in the model of financial digitization: (a) from the initial education system for certain categories of students to wider access to the categories previous excluded (less educated, from rural areas etc.), by lifelong learning programs for basic digital competencies and the promotion of minimum digitization programs for access to specific products/services, including the involvement of market actors for mass digitization; (b) from the limited access to a digitized financial system to informal asymmetric mass digitization for current needs; and (c) from minimal financial education to more specific training programs, based on a significant reorganization of the initial financial education system in two ways: decreasing the age of initiation in financial education towards lower secondary education and the autonomy of a specific discipline for all categories of specializations in the upper secondary system, not only for the economic one. At present, financial digitization is becoming the generic/basic competence necessary to prepare young people for entering the labor market and a modern form of organizing the consumption model.

The main limitation of our analysis was the lack of integrated data for 2020 in the Global Findex database, unavailable at the time of the research, but we highlighted the trend of prepandemic financial inclusion and synthesized available, disparate, partial information on developments in the first almost two years of the pandemic, estimating the possible trend for the postpandemic period with the highlighting of possible obstacles, risks. Of course, the gradual return to an activity without pandemic restrictions will determine the reconsideration of the proportion of digitization and banking of transactions for some users. It is clear that there will be no return to the precrisis situation, the positive externalities of digitized transactions have changed the behavior of individuals and companies. This is the main reason why we designed a sustainable digital financial inclusion model for the postpandemic period, as a component of economic growth and quality of life.

The partnership between the banking system—less burdensome in terms of banking costs—with local authorities and the business community in supporting access to digitized financial infrastructure and financial education will redefine the trading model at all levels. Therefore, we consider that explaining the components of interest for sustainability and resilience of the financial inclusion process will facilitate reconsideration, updating the curriculum for initial financial education, banking services and their cost for individuals and small consumers in the business environment. Financial inclusion, in order to become an instrument of robust and resilient economic recovery, should in turn be sustainable and attractive to individuals. The pandemic actually highlighted the need, but the on-the-go adaptation did not ensure an efficient and long-lasting approach to its use in post-pandemic conditions. That is why we have developed the conceptual model for promoting financial inclusion in less developed economies, which have entered the pandemic unprepared from this perspective, either in terms of legislative and institutional development or supporting policies to increase the attractiveness of transaction banking. Such an approach requires social responsibility and business ethics for the actors involved, according to the principle of convergence of long-term benefits, synthesized through resilience and sustainability.

The five pillars of the sustainable digital financial inclusion model can be the starting point for the development of an index of sustainable resilient social inclusion in the postpandemic era that better reflects the dynamics of the phenomenon. Increasing the resilience of financial inclusion implies, therefore, the redirection to the cumulative achievement of the following desiderata: (a) solid and accurate financial knowledge for the public at large and early financial education for youth, thus transforming financial education into a public good; (b) digital tools, affordable infrastructure (physical and financial infrastructure) and low-cost financial/banking services, responsibly delivered for individuals and SMEs; (c) partnership for vulnerable groups, access to help address poverty, encouragement of the growth of businesses and related employment, and boosting savings, credit, insurance, and remittances; and (d) increasing transactions’ security (enforcement of appropriate laws and regulations, including on consumer protection, building safeguards for trusted data use) and reducing the share of unbanked adults in lower-middle-income and low-income countries. Undoubtedly the COVID-19 crisis has represented “an opportunity for many countries to progress more rapidly in facilitating digital financial services, enhancing regulatory and physical infrastructure, so that service providers can meet new demand” [108,109,110], but it is necessary to create and sustain the resilience and sustainability of financial inclusion through national initiatives (national financial inclusion strategy, reforming retail and tax payments and remittances market, strengthening of financial consumer protection) and global (i.e., the World Bank’s “integrated and unified approach” focusing on nine intertwined areas in the International Financial Corporation 3.0 strategy). [116].

At the level of countries with a reduced degree of financial inclusion, (re)designing the national financial inclusion strategy could start from implementing the pillars of our conceptual model, for a responsible and inclusive strategy based on a resilience- and sustainability-building framework.

The lack of an updated database for 2020 only makes it possible to highlight only the partial impact of the pandemic. Completing the Global Findex series and resuming the analysis will allow the consolidation of some of our conclusions and a more in-depth analysis of the sociodemographic attributes of the people defined as customers of the banking system. Complementarily, the authors propose a development of research from the perspective of the analysis of financial education programs adjusted and promoted postpandemic, as an important stage of redefining the financial education model of young people.

## Figures and Tables

**Table 1:** Eigenvalue.

Communalities		
	Initial	Extraction
Account (% age 15+)	1.000	0.996
Account. male (% age 15+)	1.000	0.979
Account. in labor force (% age 15+)	1.000	0.799
Account. out of labor force (% age 15+)	1.000	0.937
Account. female (% age 15+)	1.000	0.973
Account. young adults (% ages 15–24)	1.000	0.774
Account. older adults (% ages 25+)	1.000	0.978
Account. primary education or less (% ages 15+)	1.000	0.842
Account. secondary education or more (% ages 15+)	1.000	0.945
Account. income. poorest 40% (% ages 15+)	1.000	0.982
Account. income. richest 60% (% ages 15+)	1.000	0.979
Account. rural (% age 15+)	1.000	0.985
Account. urban (% age 15+)	1.000	0.982

Extraction Method: Principal Component Analysis.

**Table 2:** Descriptive statistics.

Paired Samples Statistics							
			Statistic	Bootstrap ^a^			
				Bias	Std. Error	95% Confidence Interval	
						Lower	Upper
Pair 1	Account. male (% age 15+)	Mean	0.835763	−0.000268	0.027687	0.774476	0.886354
		N	45				
		Std. Deviation	0.1811504	−0.0033037	0.0224393	0.1300994	0.2188548
		Std. Error Mean	0.0270043				
	Account. female (% age 15+)	Mean	0.808915	−0.000258	0.030886	0.739922	0.866169
		N	45				
		Std. Deviation	0.2029120	−0.0030470	0.0211476	0.1536688	0.2370336
		Std. Error Mean	0.0302483				
Pair 2	Account. out of labor force (% age 15+)	Mean	0.731848	−0.000044	0.038549	0.645534	0.802501
		N	45				
		Std. Deviation	0.2522961	−0.0039937	0.0267163	0.1937517	0.3020290
		Std. Error Mean	0.0376101				
	Account. in labor force (% age 15+)	Mean	0.872829	−0.000563	0.030224	0.806876	0.927699
		N	45				
		Std. Deviation	0.2006518	−0.0071103	0.0457540	0.1047057	0.2837053
		Std. Error Mean	0.0299114				
Pair 3	Account. young adults (% ages 15–24)	Mean	0.670945	0.000340	0.037899	0.593027	0.742627
		N	45				
		Std. Deviation	0.2483807	−0.0037557	0.0166311	0.2124366	0.2771743
		Std. Error Mean	0.0370264				
	Account. older adults (% ages 25+)	Mean	0.849378	−0.000369	0.027906	0.787047	0.901535
		N	45				
		Std. Deviation	0.1829446	−0.0029490	0.0217086	0.1324199	0.2182820
Pair 4	Account. primary education or less (% ages 15+)	Std. Error Mean	0.0272718				
		Mean	0.698118	−0.000174	0.040618	0.613800	0.775433
		N	45				
		Std. Deviation	0.2684567	−0.0034275	0.0165081	0.2286634	0.2967826
		Std. Error Mean	0.0400192				
	Account. secondary education or more (% ages 15+)	Mean	0.863027	−0.000127	0.025887	0.806171	0.909260
		N	45				
		Std. Deviation	0.1689842	−0.0036587	0.0242915	0.1150508	0.2100197
		Std. Error Mean	0.0251907				
Pair 5	Account. income. poorest 40% (% ages 15+)	Mean	0.764705	−0.000159	0.036263	0.685704	0.832916
		N	45				
		Std. Deviation	0.2378667	−0.0034492	0.0232048	0.1848892	0.2764515
		Std. Error Mean	0.0354591				
	Account. income. richest 60% (% ages 15+)	Mean	0.8599	−0.0003	0.0247	0.8044	0.9045
		N	45				
		Std. Deviation	0.16136	−0.00295	0.02081	0.11415	0.19546
		Std. Error Mean	0.02405				
Pair 6	Account. rural (% age 15+)	Mean	0.806723	−0.000239	0.032130	0.736557	0.866465
		N	45				
		Std. Deviation	0.2097440	−0.0034435	0.0241283	0.1558651	0.2513515
		Std. Error Mean	0.0312668				
	Account. urban (% age 15+)	Mean	0.8371	−0.0003	0.0264	0.7777	0.8846
		N	45				
		Std. Deviation	0.17348	−0.00273	0.01906	0.12937	0.20446
		Std. Error Mean	0.02586				

^a^ Unless otherwise noted, bootstrap results are based on 1000 bootstrap samples.

**Table 3:** Testing the differences between groups.

Paired Samples Test									
		Paired Differences					t	df	Sig. (2-Tailed)
		Mean	Std. Deviation	Std. Error Mean	95% Confidence Interval of the Difference				
					Lower	Upper			
Pair 1	Account. male (% age 15+)—Account. female (% age 15+)	0.0268480	0.0587885	0.0087637	0.0091860	0.0445100	3.064	44	0.004
Pair 2	Account. out of labor force (% age 15+)—Account. in labor force (% age 15+)	−0.1409812	0.1302340	0.0194141	−0.1801078	−0.1018546	−7.262	44	0.000
Pair 3	Account. young adults (% ages 15–24)—Account. older adults (% ages 25+)	−0.1784335	0.1473078	0.0219593	−0.2226896	−0.1341773	−8.126	44	0.000
Pair 4	Account. primary education or less (% ages 15+)—Account. secondary education or more (% ages 15+)	−0.1649091	0.1615661	0.0240848	−0.2134489	−0.1163693	−6.847	44	0.000
Pair 5	Account. income. poorest 40% (% ages 15+)—Account. income. richest 60% (% ages 15+)	−0.0951613	0.0904219	0.0134793	−0.1223270	−0.0679955	−7.060	44	0.000
Pair 6	Account. rural (% age 15+)—urban	−0.0303414	0.0553019	0.0082439	−0.0469560	−0.0137269	−3.680	44	0.001

**Table 4:** Testing the differences between groups ANOVA.

		Sum of Squares	df	Mean Square	F	Sig.
GDP/capita	Between Groups	46,993.387	2	23,496.693	12.310	0.000
	Within Groups	80,169.595	42	1908.800		
	Total	127,162.981	44			
Primary education	Between Groups	2,027,438,281,546.901	2	1,013,719,140,773.451	1.963	0.153
	Within Groups	21,693,324,235,317.900	42	516,507,719,888.521		
	Total	23,720,762,516,864.800	44			
Secondary education	Between Groups	1,892,076,989,138.040	2	946,038,494,569.020	0.964	0.390
	Within Groups	58,502,013,437,693.070	42	1,392,905,081,849.835		
	Total	60,394,090,426,831.110	44			
Bachelor	Between Groups	458,514,789,598.221	2	229,257,394,799.111	0.423	0.658
	Within Groups	22,785,515,704,173.426	42	542,512,278,670.796		
	Total	23,244,030,493,771.650	44			
Master	Between Groups	134,536,861,533.269	2	67,268,430,766.635	1.077	0.350
	Within Groups	2,623,512,022,946.375	42	62,464,571,974.914		
	Total	2,758,048,884,479.644	44			
Doctorate	Between Groups	1,078,417,221.561	2	539,208,610.780	1.297	0.284
	Within Groups	17,463,923,954.439	42	415,807,713.201		
	Total	23,720,762,516,864.800	44			

**Table 5:** ANOVA between groups.

	Sum of Squares	df	Mean Square	F	Sig.
Between Groups	1.298	2	0.649	91.507	0.000
Within Groups	0.298	42	0.007		
Total	1.596	44			
